# Valorization of Waste Obtained from Oil Extraction in Moringa Oleifera Seeds: Coagulation of Reactive Dyes in Textile Effluents

**DOI:** 10.3390/ma7096569

**Published:** 2014-09-12

**Authors:** Mercè Vilaseca, Víctor López-Grimau, Carmen Gutiérrez-Bouzán

**Affiliations:** 1 Institute of Textile Research and Industrial Cooperation of Terrassa (INTEXTER), Universitat Politècnica de Catalunya-Barcelona Tech, Terrassa, Barcelona 08222, Spain; E-Mails: vilaseca@intexter.upc.edu (M.V.); gutierrez@intexter.upc.edu (C.G.-B.); 2 Department of Project Engineering, Universitat Politècnica de Catalunya-Barcelona Tech, Terrassa, Barcelona 08222, Spain

**Keywords:** Moringa oleifera, natural coagulant, reactive dyes, color removal, textile wastewater, water reuse, cotton dyeing

## Abstract

Moringa oleifera seeds contain about 40% of highly valued oil due to its wide range of applications, from nutritional issues to cosmetics or biodiesel production. The extraction of Moringa oil generates a waste (65%–75% of seeds weight) which contains a water soluble protein able to be used either in drinking water clarification or wastewater treatment. In this paper, the waste of Moringa oleifera extraction was used as coagulant to remove five reactive dyes from synthetic textile effluents. This waste constitutes a natural coagulant which was demonstrated to be effective for the treatment of industrial reactive dyestuff effluents, characterized by alkaline pH, high NaCl content and hydrolyzed dyes. The coagulation yield increased at high NaCl concentration, whereas the pH did not show any significant effect on dye removal. Moringa oleifera showed better results for dye removal than the conventional treatment of coagulation-flocculation with FeCl_3_ and polyelectrolyte. Treated water can be reused in new dyeing processes of cotton fabrics with high quality results.

## 1. Introduction

Moringa oleifera Lam. (synonym *Moringa pterygosperma Gaertn*.) is a tropical multipurpose tree originally growing in India, Pakistan, Asia Minor, Africa and Arabia [1] and that has been later distributed to Central America, North and South America, and the Caribbean Islands [2]. Almost every part of the plant (leaves, flowers, seeds, roots and bark) can be used as food or for medicinal and therapeutic purposes [3].

Interest in the oil extracted from Moringa oleifera seeds has grown significantly over the years. This vegetable oil, known commercially as “Ben” or “Behen” oil, is suitable for edible purposes as it contains high levels of oleic acid (closer to 80%) [4]. Apart from nutritional purposes, other applications of Ben Oil have been reported in cosmetics, lubricants for fine machinery and lately for biodiesel production [5].

The powder of Moringa oleifera seeds is also reported as coagulant/flocculant agent for drinking water clarification due to its high content of a water soluble cationic protein able to reduce turbidity [6,7]. The defatted residues generated from Moringa oleifera oil extraction can be valorized as water treatment agent, not only for drinking water clarification, but also for municipal wastewater treatment [8,9]. The use of natural coagulant/flocculant agents is especially prominent in developing countries, in substitution of external chemical coagulants (aluminum sulfate, ferric chloride) [10,11]. These natural coagulant/flocculants can also be applied in industrial wastewater treatment, as is in the case of wastewater generated in textile dyeing and finishing processes [12]. In recent years, the valorization of natural waste as low-cost materials for the treatment of textile effluents has garnered interest [13,14,15].

In this work, the use of waste from Moringa oleifera oil extraction is proposed for the removal of dyes from textile wastewater in substitution of more expensive decolorization methods which require the use of chemical reagents or higher operation and maintenance technologies, namely: coagulation/flocculation with Fe or Al salts [16,17], adsorption with active carbon [18,19], membrane technology [20,21,22,23], and advanced oxidation methods (ozonation [24,25], Fenton and Photo-Fenton reactions [26,27], heterogeneous photocatalysis [28,29] or electrochemical techniques [30,31]). In some cases, hybrid systems are applied to treat highly concentrated dyebaths, as is the case of combined process of coagulation/flocculation and nanofiltration [32].

Valorization of Moringa oleifera wastes to treat textile wastewater could be of special interest for the Asian, African and American regions where the plant is grown, as there are many countries with a strong textile sector, for example China, India, or Brazil [33].

The textile industry is characterized by the large volume and variability of wastewater produced in their dyeing and finishing processes. Worldwide, 280,000 tons of textile dyes are discharged in industrial effluents every year, being azo dyes the most widely used with more than 60% of the total dye production [34]. Azo dyes can be fragmented by reduction reactions of the azo group, generating aromatic amines. The aromatic amines can be harmful and some of them have been classified as carcinogenic and genotoxic by the International Agency for Research on Cancer [35]. The presence of toxic and carcinogenic products in textile dyeing effluents is a problem added to the high coloration inherent to this type of effluents [36].

Among the different kinds of textile dyes, reactive ones are widely used in the dyeing of cotton and other cellulosic fibers since they offer high washing fastness and brilliant colors. Reactive dyes represent 25% of the total world market [37]. On the other hand, these dyes have a relatively low degree of exhaustion and fixation, from 70% to 90% [38]. The unfixed dyes are hydrolyzed and cannot be reused because they are unable to be covalently fixed to the cellulosic fibers. Consequently, substantial amounts of unfixed dyes are released in the wastewater.

Another environmental concern associated to dyeing with reactive dyes is the generation of highly saline effluents due to the amount of electrolyte, generally NaCl, required to increase the dye exhaustion and fixation. About 50–80 g·L^−1^ of salt is added during the reactive dyeing [38] which is almost completely discharged into the wastewater after the dyeing process. The presence of high concentration of salts is very harmful for aquatic ecosystems. Moreover, its elimination is only possible through applying costly treatments as reverse osmosis [39] or electrodialysis [40]. These environmental problems draw attention to residual reactive dyebath discoloration. In addition, the reuse of discolored salt-containing effluents in new dyeing processes can also be considered. This approach entails a significant saving of water and electrolyte.

This work is focused on the use of the waste of oil extraction from Moringa oleifera seeds to eliminate five azo reactive dyes from synthetic effluents. In recent years, some studies have been published about the treatment of acid dyes (anionic dyes) with Moringa oleifera seeds [41,42] but to our knowledge there is no previous literature centered on the use of Moringa oleifera to remove reactive dyes, nor on the use of waste from seed extraction. The effect of pH, dye hydrolysis and salt content in simulated dye baths are studied with the aim to evaluate the viability of Moringa oleifera waste to treat industrial reactive dyeing effluents. The efficiency of dye removal with Moringa oleifera waste is compared with the use of the chemical coagulation-flocculation. Finally, the feasibility of reusing the treated water to carry out new dyeing of cotton fabrics was verified.

## 2. Experimental Section

### 2.1. Moringa oleifera Oil Extraction

Dry Moringa oleifera seeds were supplied by the *Centre National de Semences Forestières* of Burkina Faso. Shells were removed manually and kernels were reduced to powder using a domestic grinder (Moulinex, Groupe SEB Ibérica, Barcelona, Spain).

One g of M. oleifera crushed seeds fed to a Soxhlet extractor fitted with a 250 mL round-bottom flask and a condenser. The extraction was run for 2 h in triplicate (*n* = 3) with 100 mL of two different solvents: hexane and ethanol (Sharlau, Sharlab S.L., Sentmenat, Barcelona, Spain). After the extraction, the solvent was distilled off under vacuum in a rotary evaporator. The extracted oil yield was expressed as percentage in weight (mean ± standard deviation). Oil yield extraction was higher for ethanol (36.1% ± 1.83%) than for hexane (24.6% ± 2.19%). The ethanol extraction yield is in accordance with results of other authors [43] whereas hexane only achieves a partial oil extraction. For this reason, in this work, ethanol was selected as solvent for oil extraction.

Protein content of the Moringa oleifera seeds before and after oil extraction was determined by analysis of Nitrogen Kjeldahl (protein = N(%) × 6.25). The protein content of seeds before extraction was 25.0% ± 1.45% (mean ± standard deviation, *n* = 3). The protein content of the defatted residues after extraction with ethanol was 34.38% ± 2.03%. This increase in the protein content should favor the coagulant effect of Moringa oleifera solutions.

### 2.2. Preparation of Coagulant from Moringa oleifera

Residues obtained after the extraction of oil from the seeds, also called Moringa oleifera meal, were dried at 60 °C for 24 h and latter were kept at room temperature. In order to evaluate the influence of salt on the extraction of the coagulant protein, two different suspensions at 5% (w/v) were prepared by stirring at room temperature (for 2 h) a portion of the oilseed residue: one with distilled water (referred as Mw) and another one with NaCl 1 M (referred as Ms). In order to evaluate the influence of coagulant filtration on dye removal efficiency, each coagulant solution (Mw and Ms) was divided again in two fractions: the first one was kept to be used directly (Mwd and Msd, respectively) and the second one was filtered (Mwf and Msf) with 0.45 µm glass filter (Millipore, Madrid, Spain).

### 2.3. Preparation of Reactive Dye Solutions

**Figure 1:**
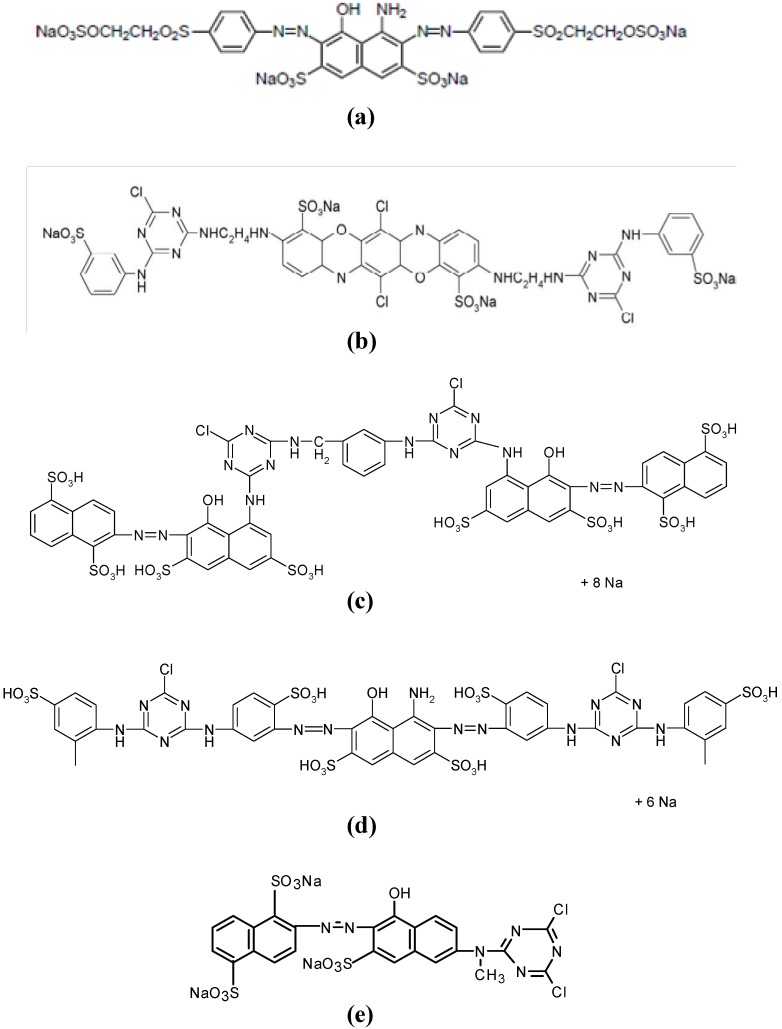
Chemical structures of the selected reactive dyes: (**a**) Remazol Black B-133 (**b**) Procion Blue H-EXL (**c**) Procion Crimson H-EXL (**c**) and (**d**) mixture: Procion Navy H-EXL (**e**) Procion Orange MX-2R.

Five azo reactive dyes widely used on cotton dyeing industries were selected in this study. They were supplied by DyStar. Their chemical structures are shown in Figure 1. In the literature, these five dyes have been the target of several studies of elimination by different technologies [29,30,44].

The Orange Procion MX-2R has the smallest structure with only one azo group as chromophore and one reactive group (dichlorotriazine). All the other dyes have two azo groups and are bi-functional. Procion H-EXL dyes have two monochlorotriazine reactive groups whereas Remazol Black dye has two vinyl sulfone groups. All dyes have several sulfonic groups in their chemical structure to increase their solubility in water. The negative charge of sulfonic groups will probably favor the coagulation of dyes with the positive charged proteins of Moringa oleifera.

Simulated dye baths were prepared at a dye concentration of 0.1 g/L in decalcified water. In the textile industry the dyeing process with reactive dyes is carried out with the addition of salt as electrolyte and at alkaline pH. This procedure generates alkaline and saline residual dye baths which contain the reactive dye in its hydrolyzed form.

The range of pH was selected from 5 to 11 (5, 7, 9 and 11) and it was adjusted with HCl 1 M or NaOH 1 M. The lower pH was established in previous published studies [41,42] and the more alkaline corresponds to the usual value in industrial effluents. The NaCl concentration in dye solutions was fixed at 60 g/L (1 M), as it is usual in dying process with reactive dyes [45].

To ensure the dye hydrolysis, all solutions were initially prepared at pH 12, boiled for 2 h and settled for 24 h. After this process, the solution was adjusted to the corresponding experimental conditions (pH and NaCl concentration).

### 2.4. Dye Removal Tests

Tests of dye removal were carried out with 50 mL dye solution samples by adding different concentrations of Moringa oleifera seed extract at ranging from 1250 to 250 mg/L of Moringa oleifera, in order to optimize this parameter.

Samples were first submitted to a fast stirring at 150 rpm for 10 min and subsequently they were stirred slowly at 20 rpm for 30 min. Finally, the treated samples were allowed to stand 24 h to promote natural decantation.

Dye removal values were calculated by UV-visible spectrophotometric measurements (Shimadzu UV-2401 PC, Shimadzu Corp., Kyoto, Japan), from the absorbance values at the maximum dye absorption wavelength (indicated in Table S1 of Supplementary Information). For each dye, the interval of linear relationship absorbance *vs.* dye concentration was previously established. All dye removal tests were carried out in quintuplicate.

Once the optimal conditions were selected (type of extraction, concentration of Moringa Oleifera and pH), trials with 1 L of dye solution samples were performed in a Jar Test which is designed for the small scale reproduction of industrial coagulation-flocculation treatments.

Conventional coagulation/flocculation tests were also carried out in Jar Test with 750 mg/L Iron (III) chloride solution (10%) and 1 mg/L polyelectrolyte TS140 (Deripol, Barcelona, Spain) solution (1 g/L) as coagulant and flocculant, respectively, which are the common values for this type of water.

For Jar Test trials, dye removal measurements were performed after 30 min (V30 min) and 24 h (V24 h) decantation times in Imhoff cones.

### 2.5. Cotton Dyeing with Decolorized Water

Samples of treated water were used for the dyeing of cotton fabrics (referred hereafter as reused dyeings) with the five selected dyes. Reference dyeings with decalcified tap water were also carried out. Dyeing tests were carried out at a liquor ratio (LR) 1/10 (fibre weight/water volume) [46] in a laboratory batch dyeing machine (Ti-Color OTX 200, Intex System, Prato, Italy) equipped with 12 stainless steel drums of 100 mL. Decolorated water was mixed and filtered with glass fiber to eliminate suspended solids. Cotton knitted fabric (Pique), scoured and prepared for dyeing, was selected.

Individual dyeing of 10 g of cotton samples was performed at standard depth with each dye at 3% o.w.f. (over weight fabric), which corresponds to 3 g/L at a LR of 1/10. A dyeing with a mixture of dyes (thricromie: Blue, Crimson and Orange) was also carried out, being the concentration of each dye 1% o.w.f. (1 g/L at LR 1/10). In the case of reference dyeings, 60 g/L NaCl was added as dyeing electrolyte. With the reused water, the addition of NaCl was not necessary. In both cases, 16 g/L Na_2_CO_3_ was added as dyeing alkali.

**Figure 2:**
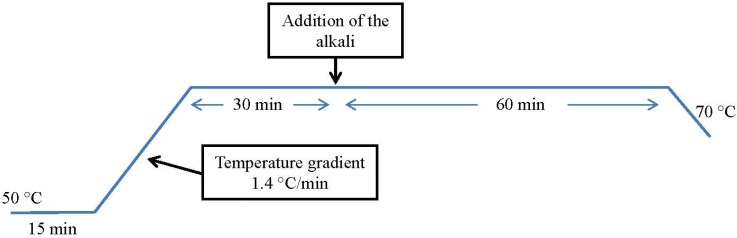
Dyeing method.

According to the information supplied by dye supplier [47], a dyeing method “all in” was selected. The dye and the entire electrolyte were included in the initial dyeing bath and the alkali was added when the dyeing process was already started (see Figure 2). All the dyeing experiments were run in triplicate.

After the dyeing process, a washing process was applied to eliminate the unfixed dye. This process consists of nine successive washing steps. The first ones (1st–3rd washes) were at 50 °C with tap water during 10 min, then a soaping step (4th wash) took place with 2 g/L of COTEMOLL TLTR at 95 °C during 15 min. After, a tap water step (5th wash) and other soap step (6th wash) were carried out with the same conditions described above. Finally, another three washes at 50 °C were carried out during 10 min (7th–9th washes). All steps were performed at liquor ratio 1:10.

Color differences between reused and reference dyeing were measured to evaluate the viability of reusing the treated effluent. With this purpose, the final dyed fabric colour was measured by a spectrophotometer Macbeth Color Eye 7000, with illuminant D65 and 10° of standard observer. The instrument evaluates the chromatic coordinates of each dyed fabric. These coordinates are defined by three parameters (Lightness *L*_cmc_; Chroma *C*_cmc_, and Hue *H*_cmc_). According to the standard UNE-EN ISO 105-J03: 1997 [48], the color difference between two samples is calculated with the formula *DE*_CMC(2:1)_, represented in the Equation (1):

*DE*_CMC(2:1)_ = [(∆L*/2*S*_L_)^2^ + (∆C*_ab_/*S*_c_)^2^ + (∆H*_ab_/*S*_H_)^2^]^1/2^ (1)

where *S*_L_, *S*_C_ and *S*_H_ are calculated from the chromatic coordinates of the standard dyeings (*L*_R_, *C*_R_ and *h*_R_) as follows:

*S*_L_ = 0.040975*L*_R_/(1 + 0.01765*L*_R_) (2)

If *L*_R_ < 16 *S*_L_ = 0.511 (3)

*S*_C_ = [0.0638*C*_R_/(1 + 0.0131*C*_R_)] + 0.638 (4)

*S*_H_ = *S*_C_(*T_f_* + 1 − *f*) (5)

*f* = {(*C*_R_)^4^/[(*C*_R_)^4^ + 1900]}^1/2^ (6)

*T* = 0.36 + │0.4·cos(35 + *h*_R_)│ if *h*_R_ ≥ 345° or *h*_R_ ≤ 164° (7)

*T* = 0.56 + │0.2·cos(168 + *h*_R_)│ if 164° < *h*_R_< 345° (8)

In general, the acceptance limit for colour differences in the textile industry is one unit (*DE*_CMC(2:1)_ ≤ 1). This criterion, which is widely used in dyeing quality control to compare the colour differences between two fabric samples, is also applied in this work.

*TOC* measurements of decolorized water ready to reuse were carried out with a *TOC* analyzer (Shimadzu *TOC*-5050A, Shimadzu Corp., Kyoto, Japan) in order to evaluate the effect of organic matter content on quality dyeing.

## 3. Results and Discussion

### 3.1. Influence of the Coagulant Solution Preparation

**Figure 3:**
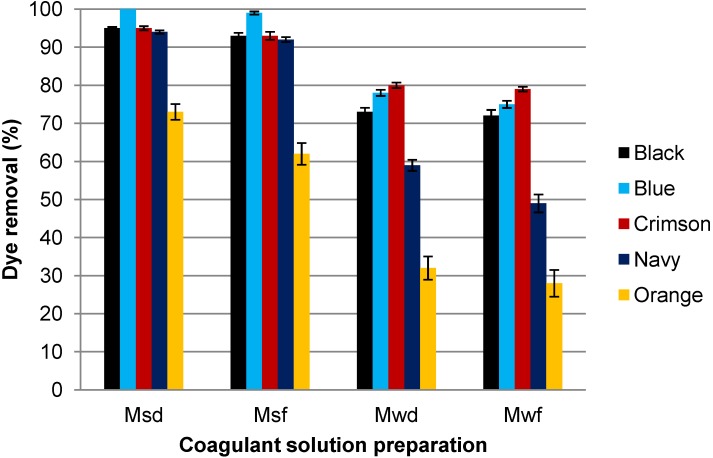
Influence of the preparation conditions of coagulant solution on dye removal efficiency (Dye 0.1 g/L; Moringa oleifera 1000 mg/L and pH 5).

Different ways to prepare the coagulant solution from Moringa oleifera suspensions were studied and their influence on dye removal efficiency was evaluated (Figure 3). These different conditions of preparation were referred as Mwd, Mwf, Msd and Msf, which correspond to the Moringa seeds waste (M) stirred in water (w) or in salt solution (s) used either directly (d) or filtered (f), as it is indicated in Section 2.2 of the Experimental Section.

Figure 3 evidences that suspensions with salt (Msd and Msf) provided higher dye removal yields than without salt (Mwd and Mwf). Thus, Msd solutions reached more than 94% of dye removal for all dye solutions, except for Orange dye, whereas Mwd solutions reached 80% of dye removal in the best of cases. The presence of salt promotes the extraction of positive charged proteins, which cause the coagulant effect of Moringa oleifera.

Also, when comparing the not filtered suspensions and the filtered solutions, it can be seen that the efficiency of the dye removal process is quite similar, although only a slight increase is shown with the not filtered suspensions.

The lower efficiency of Moringa to eliminate Orange dye with respect to the other dyes can be attributed to the smaller size of the dye molecule which only contains one reactive group. Also, the Orange MX-2R dye has three sulfonic groups whereas the new Procion H-EXL dyes have two reactive groups and 4, 6 or 8 sulfonic solubilizing groups (Table S1).

### 3.2. Influence of Dyeing Electrolyte

According to results of the previous section, Msf and Msd were selected to treat dye solutions prepared with distillated water (referred as Dye-w). However, the industrial reactive dyeing effluents are characterized by their high salt content. For this reason, in these cases the addition of salt may be unnecessary. To verify this, simulated industrial effluents were prepared with dye and 60 g/L NaCl (referred as Dye-s). Then, Dye-w solutions were treated with Moringa extracted with salt (Msf and Msd) whereas Dye-s solutions were treated with Moringa extracted in water (Mwf and Mwd). Results are displayed in Table 1.

**Table 1:** Influence of dyeing electrolyte on dye removal efficiency (Dye 0.1 g/L, Moringa oleifera 1000 mg/L, pH 5). Dye-s contains also 60 g/L NaCl.

Dye solution	Extraction	Black	Blue	Crimson	Navy	Orange
		Dye removal (%)				
Dye-w	Msf	93	98	93	92	62
	Msd	95	100	95	94	73
Dye-s	Mwf	93	94	91	90	63
	Mwd	95	97	93	95	68

Dye removal results for salt containing simulated industrial effluents were similar to the ones of dye solutions treated with Moringa extracted with salt. This fact implies that in dye solutions which already contain salt (Dye-s), the extraction of Moringa oleifera waste can be performed only with water.

Dye removal yields higher than 90% were achieved for all dyes, except for Orange Procion dye, as expected. By another hand, not filtered suspensions of Moringa oleifera (Msd and Mwd) provided again slightly better results than filtered solutions (Msf and Mwf).

For this reason in the subsequent sections, Moringa oleifera suspensions were prepared only with water and were used directly without filtration (Mwd). Salt was added to the synthetic dye effluent to simulate industrial reactive dyebaths.

### 3.3. Influence of the pH

**Figure 4:**
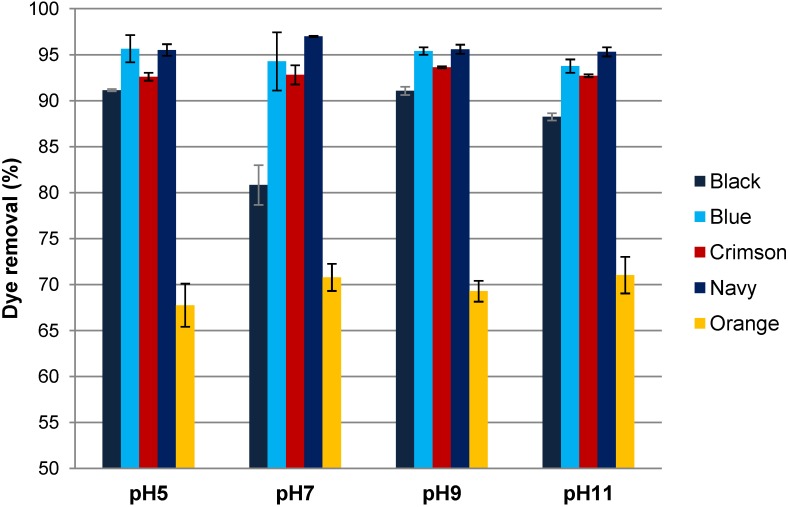
Influence of pH on dye removal efficiency (0.1 g/L dye, 60 g/L NaC, Moringa oleifera 1000 mg/L).

Different pH values ranging from pH 5–11 were tested in order to evaluate their influence on the dye removal efficiency (Figure 4).

As is shown in Figure 4, dye removal results were almost independent of pH, being slightly better at pH 9 for Black, Blue and Crimson. At this pH, dye removal values were higher than 90% for all dyes except for the Orange, which in all cases obtained lower results (closer to 70% of dye removal).

As reactive dyeing effluents and the subsequent washing baths are alkaline, this fact facilitates industrial application because pH adjustment is not required.

### 3.4. Influence of Coagulant Concentration

**Figure 5:**
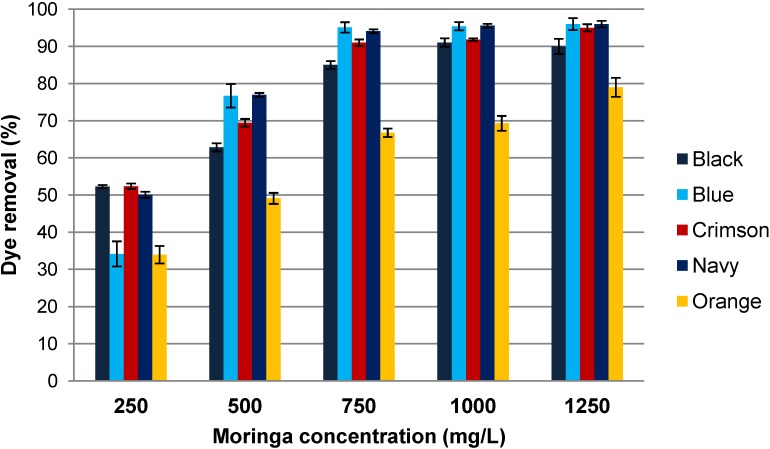
Influence of Moringa oleifera concentration on dye removal (0.1 g/L dye, 60 g/L NaCl and pH 9).

In order to optimize the addition of coagulant to the dye solutions, several Moringa oleifera suspensions were prepared in a range of 250 mg/L–1250 mg/L. Dye removal results obtained are shown in Figure 5.

Figure 5 evidences the increase of dye removal when Moringa oleifera concentration was also increased up to 750 mg/L. More than 90% of dye removal was achieved with the 750 mg/L solutions for Blue, Crimson and Navy dye solutions and more than 85% for Black dye solution. The Black dye solution reached 90% of dye removal with the addition of 1000 mg/L Moringa solutions. Orange dye is the most difficult to be coagulated with Moringa oleifera. Nevertheless, the addition of 1250 mg/L Moringa solution enabled obtaining Orange dye removal close to 80%.

### 3.5. Efficiency of Moringa oleifera Waste *versus* Ferrous (III) Chloride

The dye removal efficiency of Moringa oleifera waste was compared with respect to the conventional wastewater coagulation-flocculation method with Ferrous (III) Chloride and a polyelectrolyte. In both cases, coagulant concentration was fixed at 750 mg/L. Jar Tests trials were carried out with 1 L samples which were subsequently allowed to settle in Imhoff cones. Dye removal and settled sludge volumes were determined after 30 min (V30 min) and 24 h (V24 h) decantation times.

As can be seen in Table 2, Moringa oleifera waste is much more efficient on dye removal than the conventional coagulation/flocculation treatment with FeCl_3_ and polyelectrolyte. Moringa oleifera reached 89%–96% removal yield for Blue, Crimson and Navy dyes after 30 min decantation and 96%–97% after 24 h. For the Black dye, the removal yield was 85% and for the Orange it decreased to 53%. In opposite, the dye removal obtained with 750 mg/L FeCl_3_ and 1 mg/L polyelectrolyte was very low, ranging from 0% to 56%. Only the Blue dye surpassed 50% removal yield.

**Table 2:** Jar Tests trials with Moringa oleifera waste (750 mg/L) and with FeCl_3_ (750 mg/L) + polyelectrolyte (1 mg/L). Dye 0.1 g/L, NaCl 60 g/L and pH 9. Decantation time: 30 min (V30 min) and 24 h (V24 h).

Jar test		Black	Blue	Crimson	Navy	Orange
Dye removal (%)						
Moringa	V30 min	83	90	89	96	54
	V24 h	85	96	96	97	53
FeCl_3_ + poly	V30 min	9	56	24	30	3
	V24 h	4	53	20	25	0
Settled sludge volume (mL/L)						
Moringa	V30 min	38	150	100	130	33
	V24 h	27	60	40	59	29

In this way, the promising results obtained with Moringa oleifera in the previous sections were corroborated by Jar Tests. Thus, whereas dye removal results with FeCl_3_ and polyelectrolyte were very poor, high dye removal rates were obtained with Moringa oleifera after 30 min decantation, which were even increased after 24 h decantation.

In addition, it should be highlighted that sludge generated by Moringa oleifera waste was easy to be decanted and settled. In fact, high settled sludge volumes (100–150 mL/L) were generated after 30 min of decantation in the three dye solutions with almost full dye removal (Blue, Crimson and Navy). These values were significantly decreased to 40–60 mL/L when decantation was extended to 24 h.

### 3.6. Reuse of the Decolorized Effluent for New Dyeing Process

Cotton fabrics were dyed with the five selected dyes with the decolorized water in order to evaluate the feasibility in reusing these high salt containing effluents. Dyeing was performed with a single dye and also with a trichromie of dyes (mixture of Blue, Crimson and Orange), as trichromies are very common in the textile dyeing process [49]. Color differences of each sample were evaluated with respect to the corresponding reference dyeing made with clean water (Table 3).

As can be seen in Table 3, color differences (*DE*_CMC(2:1)_) below 0.5 were obtained in dyeing with Blue, Crimson and Navy dyes, as well as for the trichromie, which is an excellent result. On the contrary, for Black and Orange dyes, color differences were slightly above the limit established in quality dyeing control (*DE*_CMC(2:1)_ ≤ 1).

The dyeing color differences, the dye removal rate and *TOC* content of the decolorized water reclaimed in the dyeing can be compared in Table 4. High quality dyeing with very low color differences were obtained when the residual dye was almost removed. On the other hand, color differences were higher if the residual hydrolyzed dye was not properly removed. The residual hydrolyzed dye decreases the fixation of the new dye. Although the former cannot react with the cotton fiber, it can occupy the sites for the fixation of the new dye and in this way, the dye fixation rate is slowed down which implies an increase of new dye hydrolysis.

**Table 3:** Color differences of dyeing performed with decolorized water with respect to the corresponding reference dyeing.

Dyeing	*DL*	*DC*	*DH*	*SL*	*SC*	*SH*	*DE*_CMC(2:1)_
Black	−1.35	−0.59	0.27	0.67	1.79	1.18	1.09
Blue	0.22	0.13	−0.34	0.96	4.04	2.45	0.18
Crimson	0.54	−0.11	−0.66	0.89	6.65	4.52	0.33
Navy	−0.72	0.10	−0.01	0.73	2.02	1.30	0.49
Orange	2.28	−5.40	−1.49	1.15	9.04	3.90	1.22
Trichromie	0.37	−0.15	0.06	0.71	1.79	1.18	0.27

An important fact to be highlighted is that the presence of organic matter in the reused water due to Moringa oleifera treatment did not cause any effect on the quality of dyeing. Very small color differences were obtained with reused water which contained 91–97 mg/L of *TOC*.

**Table 4:** Dyeing color differences and characteristics of the reused water (dye removal rate and *TOC* content).

Parameter	Black	Blue	Crimson	Navy	Orange
Dye Removal (%)	85	96	96	97	53
*TOC* (mg/L)	101.1	95.9	91.4	97.4	108.0
*DE*_CMC(2:1)_	1.09	0.18	0.33	0.49	1.22

## 4. Conclusions

The defatted Moringa oleifera wastes can be valorized as coagulant solutions for the removal of dyes from textile wastewater.

The optimal Moringa oleifera waste concentration was 750 mg/L. The coagulant effect of Moringa solutions/suspensions is enhanced in the presence of NaCl, which is used as dyeing electrolyte. Dye removal results were almost independent on pH, although they were slightly better at pH 9 (the usual pH of reactive dyeing and washing effluents). These conditions provided higher dye removal yield than with use of the conventional coagulation-flocculation with FeCl_3_ and polyelectrolyte.

For a typical residual reactive dyebath, more than 95% Blue, Crimson and Navy dyes are removed with Moringa oleifera waste, 85% Black dye and almost 80% of Orange dye.

The different behavior of dyes with respect to the Moringa oleifera coagulant action can be attributed to their chemical structure, and specifically to the number of sulfonic groups. The Orange dye is the smallest one with only three sulfonic groups whereas the rest of dyes have four, six or eight sulfonic groups in their molecular structure.

Finally, the feasibility of reusing decolorized water in new dyeing processes with reactive dyes was demonstrated. The reuse of decolorized dye baths not only entails water saving but also NaCl saving and the reduction of salinity in the discharged wastewater. Very good quality dyeing with *DE*_CMC(2:1)_) below 0.5 were obtained for Blue, Crimson and Navy dyes as well as for the trichromie. For Black and Orange dyes, color differences were slightly above the limit established in quality dyeing control (*DE*_CMC(2:1)_ ≤ 1).

## Supplementary Information

**Table S1:** Characteristics of the five reactive dyes studied.

Commercial Name	C.I. Name	Chromophore/reactive group	Number of sulfonic groups	λ max (nm)	Chemical structure (Figure 1)
Remazol Black B-133	Reactive Black 5	Bis-azo/bis-vinyl sulfone	4	599	a
Procion Blue H-EXL	Reactive Blue 198	Bis-oxazine/bis-monochlorotriazine	4	625	b
Procion Crimson H-EXL	Reactive Red 231	Bis-azo/bis-monochlorotriazine	8	546	c
Procion Navy H-EXL	Not available	Bis-azo/bis-monochlorotriazine	8 or 6	606	c and d (mixture)
Procion Orange MX-2R	Reactive Orange 4	Azo/dichlorotriazine	3	490	e

## References

[1] Mughal M.H., Ali G., Srivastava P.S., Iqbal M. (1999). Improvement of drumstick (*Moringa pterygosperms Gaetn.*)—A unique source of food aand medicine through tissue culture. Hamdard Med..

[2] Oliveira J.T.A., Silveira S.B., Vasconcelos K.M., Cavada B.S., Moreira R.A. (1999). Compositional and nutritional attributes of sedes from the multiple purpose tree *Moringa oleifera* Lamarck. J. Sci. Food Agric..

[3] Anwar F., Latif S., Ashraf M., Gilani A.H. (2007). Moringa oleifera: A food plant with multiple medicinal uses. Phytother. Res..

[4] Anwar F., Bhanger M.I. (2003). Analytical characterization of Moringa oleífera seed oil grown in temperate regions of Pakistan. J. Agric. Food Chem..

[5] Rashid U., Anwar F., Moser B.R., Knothe G. (2008). Moringa Oleifera oil: A possible source of biodiesel. Bioresour. Technol..

[6] Ndabigengesere A., Narasiah K.S. (1998). Quality of water treated by coagulation using Moringa oleifera seeds. Water Res..

[7] Pritchard M., Craven T., Mkandawire T., Edmondson A.S., O’neill J.G. (2010). A study of the parameters affecting the effectiveness of Moriga oleífera in drinking water purification. Phys. Chem. Earth.

[8] Yin C.Y. (2010). Emerging usage of plant-based coagulants for water and wastewater treatment. Process Biochem..

[9] Bhuptawat H., Folkard G.K., Chaudhari S. (2007). Innovative physico-chemical treatment of wastewater incorporating Moringa Oleifera seed coagulant. J. Hazard. Mater..

[10] McConnachie G.L., Folkard G.K., Mtawali M.A., Sutherland J.P. (1999). Field trials of appropriate hydraulic flocculation processes. Water Res..

[11] Pritchard M., Craven T., Mkandawire T., Edmondson A.S., O’neill J.G.A. (2010). A comparison between Moringa oleifera and chemical coagulants in the purification of drinking water—An alternative sustainable solution for developing countries. Phys. Chem. Earth.

[12] Verma A.K., Dash R.D., Bhunia P. (2012). A review on chemical coagulation/flocculation technologies for removal of colour from textile wastewaters. J. Environ. Manag..

[13] Kyzas G.Z. (2012). A decolorization technique with spent “Greek Coffee” grounds as zero-cost adsorbents for industrial textile wastewaters. Materials.

[14] Kyzas G.Z., Fu J., Matis K.A. (2013). The change from past to future for adsorbent materials in treatment of dyeing wastewaters. Materials.

[15] Kyzas G.Z., Kostoglou M. (2014). Green adsorbents for wastewaters: A critical review. Materials.

[16] Carvalho G., Delée W., Novais J.M., Pinheiro H.M. (2002). A factorially-designed study of physicochemical reactive dye colour removal from simulated cotton textile processing wastewaters. Color. Technol..

[17] Golob V., Vinder A., Simonic M. (2005). Efficiency of the coagulation/flocculation method for the treatment of dyebath effluents. Dyes Pigm..

[18] Shende R.V., Mahajani V.V. (2002). Wet oxidative regeneration of activated carbon loaded with reactive dye. Waste Manag..

[19] Quan X., Liu X., Bo L., Chen S., Zhao Y., Cui X. (2004). Regeneration of acid orange-7 exhausted granular activated carbons with microwave irradiation. Water Res..

[20] Qin J.J., Oo M.H., Kekre K.A. (2007). Nanofiltration for recovering wastewater from a specific dyeing facility. Sep. Purif. Technol..

[21] Petrinic I., Andersen N.P., Sostar-Turk S., le Marechal A.M. (2007). The removal of reactive dye printing compounds using nanofiltration. Dyes Pigm..

[22] Sun S.P., Hatton T.A., Chan S.Y., Chung T.S. (2012). Novel thin-film composite nanofiltration hollow fiber membranes with double repulsion for effective removal of emerging organic matters from water. J. Membr. Sci..

[23] Ong Y.K., Li F.Y., Sun S.P., Zhao B.W., Liang C.Z., Chung T.S. (2014). Nanofiltration hollow fiber membranes for textile wastewater treatment: Lab-scale and pilot-scale studies. Chem. Eng. Sci..

[24] De Faria L.A., Santana M.H.P., da Silva L.M., Freitas A.C., Boodts J.F.C., Fernandes K.C. (2009). Application of electrochemically generated ozone to the discoloration and degradation of solutions containing the dye Reactive Orange 112. J. Hazard. Mater..

[25] Wu J., Doan H., Upreti S. (2008). Decolorization of aqueous textile reactive dye by ozone. Chem. Eng. J..

[26] Papic S., Vujevic D., Koprivanac N., Sinko D. (2009). Decolourization and mineralization of commercial reactive dyes by using homogeneous and heterogeneous Fenton and UV/Fenton processes. J. Hazard. Mater..

[27] Li Y., Zhou T., Lu X., Wang J., Wong F.-S. (2009). Rapid decolorization and mineralization of simulated textile wastewater in a heterogeneous Fenton like system with/without external energy. J. Hazard. Mater..

[28] Muruganandham M., Swaminathan M. (2006). Photocatalytic decolourisation and degradation of Reactive Orange 4 by TiO_2_-UV process. Dyes Pigm..

[29] Visa T., Sanchez M., López-Grimau V., Navarro R., Reche S., Gutiérrez-Bouzán M.C. (2012). Photocatalysis with titanium dioxide to remove colour of exhausted reactive dyebaths without pH modification. Desalin. Water Treat..

[30] Lopez-Grimau V., Gutierrez M.C. (2006). Decolourisation of simulated reactive dyebath effluents by electrochemical oxidation assisted by UV light. Chemosphere.

[31] Morsi M.S., Al-Sarawy A.A., Shehab El-Dein W.A. (2011). Electrochemical degradation of some organic dyes by electrochemical oxidation on a Pb/PbO_2_ electrode. Desalin. Water Treat..

[32] Liang C.Z., Sun S.P., Li F.Y., Ong Y.K., Chung T.S. (2014). Treatment of highly concentrated wastewater containing multiple syntethic dyes by a combined process of coagulation/flocculation and nanofiltration. J. Membr. Sci..

[33] Chemical Insight & Forecasting: IHS Chemical Chemical Economics Handbook: Dyes. http://www.ihs.com/products/chemical/planning/ceh/dyes.aspx.

[34] Solis M., Solis A., Perez H.I., Manjarrez N., Flores M. (2012). Microbial decolouration of azo dyes: A review. Process Biochem..

[35] International Agency for Research on Cancer (IARC) (2004). Overall Evaluations of Carcinogenicity to Humans. IARC Monographs.

[36] López-Grimau V., Riera-Torres M., Lopez-Mesas M., Gutierrez-Bouzan C. (2013). Removal of aromatic amines and decolourisation of azo dye baths by electrochemical treatment. Color. Technol..

[37] Carneiro P.A., Osugi M.E., Fugivara C.S., Boralle N., Furlan M., Zanoni M.V. (2005). Evaluation of different electrochemical methods on the oxidation and degradation of Reactive Blue 4 in aqueous solution. Chemosphere.

[38] López-Grimau V., Gutiérrez-Bouzán C., Fu J. (2013). Selection of decolorization methods of reactive dye baths for reuse purposes. Dyeing: Processes, Techniques and Applications.

[39] Greenlee L.F., Lawler D.F., Freeman B.D., Marrot B., Moulin P. (2009). Reverse osmosis desalination: Water sources, technology and today’s challenges. Water Res..

[40] Chao Y.M., Liang T.M. (2008). A feasibility study of industrial wastewater recovery using electrodialysis reversal. Desalination.

[41] Beltran-Heredia J., Sanchez-Martin J. (2008). Azo dye removal by Moringa oleífera seed extract coagulant. Color. Technol..

[42] Beltran-Heredia J., Sanchez-Martin J., Delagado-Regalado A., Jurado-Bustos C. (2009). Removal of Alizarin Violet 3R (anthraquinonic dye) from aqueous solutions by natural coagulants. J. Hazard. Mater..

[43] Ortiz-Palafox J., Navarrete A., Sacramento-Rivero J.C., Rubio-Atocha C., Acereto-Escoffie P., Rocha-Uribe J.A. (2012). Extraction and characterization of oil from Moringa Oleifera using supercritical CO_2_ and traditional solvents. Am. J. Anal. Chem..

[44] Kyzas G.Z., Travlou N.A., Kalogirou O., Deliyanni E.A. (2013). Magnetic graphene oxide: Effect of preparation route on Reactive Black 5 adsorption. Materials.

[45] European Commission. Integrated Pollution Prevention and Control (IPPC) Reference Document on Best Available Techniques for the Textile Industry. http://eippcb.jrc.ec.europa.eu/reference/BREF/txt_bref_0703.pdf.

[46] Lacasse K., Baumann W. (2004). Textile Chemicals. Environmental Data and Facts.

[47] DyStar, Inc. (2004). Work Procedures. Industrial Washing Procedure, Excel Washing.

[48] AENOR (Spanish Association for Standardization and Certification) (1997). Textiles. Ensayos de solidez del Color: Cálculo de las Diferencias de Color.

[49] Christie R.M. (2007). Environmental Aspects of Textile Dyeing.

